# Potential of 2-Hydroxy-3-Phenylsulfanylmethyl-[1,4]-Naphthoquinones against *Leishmania* (*L.*) *infantum*: Biological Activity and Structure-Activity Relationships

**DOI:** 10.1371/journal.pone.0105127

**Published:** 2014-08-29

**Authors:** Erika G. Pinto, Isabela O. Santos, Thomas J. Schmidt, Samanta E. T. Borborema, Vitor F. Ferreira, David R. Rocha, Andre G. Tempone

**Affiliations:** 1 Centre for Parasitology and Mycology, Instituto Adolfo Lutz, São Paulo, SP, Brazil; 2 Instituto de Medicina Tropical de São Paulo, Universidade de São Paulo, São Paulo, SP, Brazil; 3 Universidade Federal Fluminense, Instituto de Química, Departamento de Química Orgânica, Niterói, RJ, Brazil; 4 Institute of Pharmaceutical Biology and Phytochemistry (IPBP), University of Münster, PharmaCampus, Münster, Germany; University of Edinburgh, United Kingdom

## Abstract

Naphtoquinones have been used as promising scaffolds for drug design studies against protozoan parasites. Considering the highly toxic and limited therapeutic arsenal, the global negligence with tropical diseases and the elevated prevalence of co-morbidities especially in developing countries, the parasitic diseases caused by various *Leishmania* species (leishmaniasis) became a significant public health threat in 98 countries. The aim of this work was the evaluation of antileishmanial *in vitro* potential of thirty-six 2-hydroxy-3-phenylsulfanylmethyl-[1,4]-naphthoquinones obtained by a three component reaction of lawsone, the appropriate aldehyde and thiols adequately substituted, exploiting the *in situ* generation of *o*-quinonemethides (*o*-QM) via the Knoevenagel condensation. The antileishmanial activity of the naphthoquinone derivatives was evaluated against promastigotes and intracellular amastigotes of *Leishmania* (*Leishmania*) *infantum* and their cytotoxicity was verified in mammalian cells. Among the thirty-six compounds, twenty-seven were effective against promastigotes, with IC_50_ values ranging from 8 to 189 µM; fourteen compounds eliminated the intracellular amastigotes, with IC_50_ values ranging from 12 to 65 µM. The compounds containing the phenyl groups at R_1_ and R_2_ and with the fluorine substituent at the phenyl ring at R_2_, rendered the most promising activity, demonstrating a selectivity index higher than 15 against amastigotes. A QSAR (quantitative structure activity relationship) analysis yielded insights into general structural requirements for activity of most compounds in the series. Considering the *in vitro* antileishmanial potential of 2-hydroxy-3-phenylsulfanylmethyl-[1,4]-naphthoquinones and their structure-activity relationships, novel lead candidates could be exploited in future drug design studies for leishmaniasis.

## Introduction

Leishmaniasis is a complex of diseases caused by protozoan parasites of the genus *Leishmania*. Leishmaniasis is still one of the most neglected diseases, affecting the poorest population in developing countries [1]. The disease is endemic in 98 countries, with a global incidence estimated at approximately 0.9–1.6 million cases occurring each year and Brazil is among the 10 countries with the highest estimated case counts [2]. Currently, the leishmaniasis treatment is based on the use of few drugs such as pentavalent antimonials, miltefosine and amphotericin B, with medium to severe toxic side effects and long-term administration [3]. Over the past years, the lack of new medicines targeting parasitic diseases affecting people in developing countries has become a global concern [4]. Therefore, an urgent need remains for safer and more effective drug candidates.

Naphthoquinones are a class of chemical compounds exhibiting a variety of anticancer [5], antiviral, trypanocidal, immunomodulatory and antimicrobial activities [6]. Among the 1,4-naphthoquinone derivatives in literature, some promising antimalarials have been described [7]–[9]. Some other series of 1,4-naphthoquinone derivatives have also been widely evaluated against *Mycobacterium tuberculosis* [10], *Plasmodium falciparum* [9], and as molluscicidal candidates against *Biomphalaria glabrata* [11]. A number of reports have also shown the antiprotozoal potential of 1,4-naphthoquinone derivatives against *Trypanosoma cruzi* and *Leishmania* (*L.*) *donovani* [12]–[13]. In the present study, a series of thirty-six 2-hydroxy-3-phenylsulfanylmethyl-[1,4]-naphthoquinones (**1–36**) was synthesized and evaluated against the extracellular and the clinically relevant form of the parasite, the intracellular amastigotes of *L.* (*L.*) *infantum*, which is the etiologic agent of visceral leishmaniasis in Brazil and the Mediterranean region. The *in vitro* toxicity of these compounds against mammalian cells was also studied to provide the selectivity index. Finally, a QSAR analysis was conducted in order to obtain insights into the structural requirements for activity and selectivity within this series of naphthoquinone derivatives.

## Materials and Methods

### General Procedures

**Figure 1:**
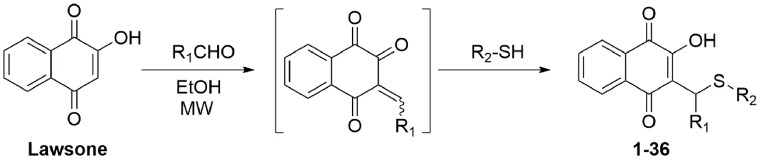
General scheme for preparing the 2-hydroxy-3-alkyl[1,4]naphthoquinones or 3-arylsulfanylmethyl[1,4]naphthoquinones under microwave irradiation.

The compounds **1–36** were obtained by reaction of lawsone with formaldehyde *in situ* generating the intermediate ortho-quinonemethide, followed by nucleophilic addition of thiols adequately substituted (Figure 1). This reaction explores *in situ* generation of *o*-quinonemethides (*o*-QM) via the Knoevenagel condensation reaction between lawsone and formaldehyde [9].

### Ethics Statement: Bioassay Procedures

BALB/c mice and Golden hamsters (*Mesocricetus auratus*) were supplied by the Animal Breeding Facility at the Adolfo Lutz Institute of São Paulo. They were maintained in sterilized cages, receiving water and food *ad libitum*, under climate-controlled (22°C±2 and relative humidity- 60%) and photoperiod-controlled (12 h light-dark cycles) environment. To avoid pain, animals were euthanized using carbon dioxide (purity 99.99%) in a gas chamber, in a flow rate of 20% of the chamber volume per minute, according to the Newcastle Consensus Meeting on Carbon Dioxide Euthanasia of Laboratory Animals (http://www.nc3rs.org.uk/downloaddoc.asp?id=416&page=292&skin=0). Animal procedures were performed with the approval of the Research Ethics Commission of Instituto Adolfo Lutz/Instituto Pasteur (project CEUA-IAL/Pasteur 04/2011) in agreement with the Guide for the Care and Use of Laboratory Animals from the National Academy of Sciences (http://www.nas.edu) and all efforts were made to minimize suffering.

### Parasites and Macrophages Maintenance

*L*. (*L.*) *infantum* (MHOM/BR/1972/LD) promastigotes were maintained in M-199 medium supplemented with 10% fetal bovine serum (FBS) and 0.25% hemin at 24°C. *L.* (*L.*) *infantum* was maintained in golden hamsters for up to approximately 60–70 days post-infection. The amastigotes were obtained from the spleens of previously infected hamsters by differential centrifugation. The macrophages were collected from the peritoneal cavity of BALB/c mice by washing with RPMI-1640 (without phenol red and supplemented with 10% FBS). NCTC (clone 929) murine conjunctive cells were maintained in RPMI-1640 (without phenol red and supplemented with 10% FBS) at 37°C in a humidified containing 5% CO_2_.

### Determination of the Antileishmanial Activity

To determine the 50% inhibitory concentration (IC_50_) against *L.* (*L.*) *infantum*, drugs were dissolved previously in dimethyl sulphoxide (DMSO) and diluted with M-199 medium. Promastigotes were counted in a Neubauer hemocytometer and seeded at 1×10^6^ cells per well in 96-well microplates using miltefosine as standard drug. The tested compounds were incubated at the highest concentration of 200 µM for 48 h at 24°C. Parasite viability was determined using the MTT assay [14]. Briefly, 3-[4,5-dimethylthiazol-2-yl]-2,5-diphenyltetrazolium bromide (MTT) was dissolved in phosphate-buffered saline (PBS) at 5 mg/mL, sterilized through 0.22 µm membranes and incubated with cells (20 µL/well) for 4 h at 24°C. The extraction of the mitochondrial formazan was done with 80 µL of 10% SDS for 18 h at 24°C. The optical density was determined in FilterMax F5 (Molecular Devices) at 570 nm. Promastigotes incubated without compounds were used as the viability control (100% viability) and without cells (blank).

The activity against intracellular *L.* (*L.*) *infantum* amastigotes was determined in infected macrophages. Macrophages were obtained as previously described and seeded for 24 h at 1×10^5^ cells/well in 16-well slide chambers (Nunc). Amastigotes were isolated from the spleens of previously infected hamsters, separated by differential centrifugation and added to the macrophages at a ratio of 1∶10 (macrophage/amastigotes) for 24 h at 37°C. Non-internalized parasites were removed by washing once with medium and the cells were then incubated with the test compounds for 120 h at 37°C in 5% CO_2_, using miltefosine as standard drug. At the end of the assay, the cells were fixed in methanol, stained with Giemsa and observed under a light microscope to determine the number of intracellular parasites. The number of amastigotes was determined in 400 macrophages from the drug-treated and control wells [15].

### Cytotoxicity against Mammalian Cells

The 50% cytotoxic concentration (CC_50_) was determined in NCTC clone 929 murine conjunctive cells. NCTC cells were seeded at 6×10^4^ cells/well in 96-well microplates at 37°C in a 5% CO_2_. The mammalian cells were incubated with tested compounds to the highest concentration of 200 µM for 48 h at 37°C, using miltefosine as standard drug. The viability of the cells was determined by MTT assay at 570 nm [15]. The selectivity index (SI) was determined considering the following equation: CC_50_ NCTC cells/IC_50_ amastigotes.

### Statistical Analysis

The obtained data represent the mean and standard deviation of two independent experiments performed in duplicate. The IC_50_ and CC_50_ values were calculated using sigmoid dose-response curves performed using GraphPad Prism version 5.0 (GraphPad Software, San Diego, CA, USA), and the 95% confidence intervals (95% CI) were included. The ANOVA test was performed to evaluate the significance (p<0.05) of data.

### QSAR Analysis

The biological activity data (IC_50_ values against *L.* (*L.*) *infantum* promastigotes available for twenty-seven compounds) were transformed to a molar dimension and used for the QSAR analysis in form of their negative decadic logarithms (pIC_50_) [16]. These values were in a range from 3.72 to 5.09. 3D molecular models of these twenty-seven compounds were generated with the modeling package MOE rel. 2011.10 (CCG, Montreal). For each structure, a conformational search was performed using a low-mode molecular dynamics method (default parameters, MMFF94X force field) as implemented in MOE. The lowest energy conformer was then energy minimized using the AM1 Hamiltonian as included in MOE's MOPAC module. The optimized geometries thus obtained were used to calculate molecular descriptors. In this study, a total of 308 descriptors were taken into consideration, i.e. analyzed for linear correlation with the target value, pIC_50_. The full descriptor table is available from the authors (TJS) on request. Descriptor selection was performed with a genetic algorithm – multiple linear regression method (GA-MLR) as available in the MOE program GA.svl [available for MOE users on the CCG SVL exchange site, http://www.chemcomp.com/Support-SVL_Exchange.htm]. This algorithm was applied to the data set using three different descriptor blocks such that (a) only 2D descriptors (n = 186) (b) only 3D descriptors (n = 122) and (c) all 308 descriptors were taken into account. For each setting, three GA runs (population size = 100) were performed, in which the allowed number of descriptors was 3, 4 and 5. The optimization criterion for the GA was a minimization of the LOF (lack of fit), the predefined maximum number of generations was 50.000 and the termination criterion was a failure to achieve an improvement (i.e. further decrease) of the LOF within 1000 generations. After termination, the final model population of each run was validated using leave-one-out cross validation and the model with the highest squared correlation coefficient (Q^2^) between the cross validation predictions and the experimental biological data was chosen for further evaluation. This was achieved by applying partial least squares (PLS) regression to the best descriptor combinations found by the GA-MLR calculations. The best model found in this way is described by equation (1).

## Results

### Antileishmanial Activity

The antileishmanial activity of the thirty-six naphthoquinone derivatives was evaluated against promastigotes of *L.* (*L.*) *infantum* by MTT reduction assay, as shown in Table 1. After 48 h of incubation, twenty-seven compounds were active with IC_50_ values in the range of 8.09 to 189.91 µM; compound **32** was the most active. According to the colorimetric assay of MTT and light microscopy, the active compounds killed 100% of parasites at the highest tested concentration. When tested against the intracellular amastigotes, fourteen compounds resulted in IC_50_ values in the range of 12.98 to 65.52 µM. Miltefosine was used as a standard drug and resulted in IC_50_ values of 16.85 and 17.80 µM, respectively. Among the tested compounds, compound **11** was the most effective, with an IC_50_ value of 12.98 µM against intracellular amastigotes. Compounds **11**, **20** and **21** showed a similar effectiveness to the standard drug miltefosine. Other tested compounds (**2**, **3**, **4**, **5**, **6**, **7**, **12**, **19**, **25**, **26**, **27**, **28** and **34**) were effective against promastigotes, but exhibited no activity against intracellular amastigotes at the highest tested concentration. In contrast, the naphthoquinone derivatives (**1**, **9**, **13**, **14**, **15**, **24**, **31**, **35** and **36**) showed no activity (>200 µM) against both forms of *L.* (*L.*) *infantum*.

**Table 1:** Antileishmanial and cytotoxicity effects of 2-hydroxy-3-phenylsulfanylmethyl-[1,4]-naphthoquinones.

*Compounds*	*R1*	*R2*	Promastigotes	Amastigotes	Cytotoxicity	S.I.
			IC_50_ (µM)	IC_50_ (µM)	CC_50_ (µM)	
			(C.I.95%)	(C.I.95%)	(C.I.95%)	
1	H	4-CH_3_C_6_H_4_	na	na	89.09 (82.77–95.88)	nd
2	H	-C_6_H_5_	124.3 (115.8–133.3)	na	84.66 (77.02–93.05)	nd
3	H	4-OCH_3_C_6_H_4_	75.0 (71.14–79.08)	na	92.57 (90.14–95.05)	nd
4	H	4-ClC_6_H_4_	85.38 (78.54–92.82)	na	82.20 (74.47–90.72)	nd
5	H	4-FC_6_H_4_	129.0 (115.6–143.90)	na	97.94 (85.80–111.80)	nd
6	H	4-CH_3_SC_6_H_4_	188.5 (177.2–200.50)	na	100.50 (86.81–116.30)	nd
7	H	4-NO_2_C_6_H_4_	8.44 (4.315–16.51)	na	85.51 (79.36–92.14)	nd
8	H	4-OHC_6_H_4_	189.91 (178.4–203.2)	65.52 (61.10–70.26)	173.62 (208.6–462.4)	2.64
9	H	Propyl	na	na	>200	nd
10	C_6_H_5_	4-ClC_6_H_4_	20.72 (17.73–24.21)	40.37 (13.16–123.80)	>200	>4.95
11	C_6_H_5_	4-FC_6_H_4_	28.82 (26.57–31.26)	12.98 (8.59–19.58)	>200	>15.4
12	C_6_H_5_	4-CH_3_SC_6_H_4_	45.10 (40.72–49.95)	na	138.2 (118.4–161.4)	nd
13	H	2-CH_3_C_6_H_4_	na	na	>200	nd
14	H	3-CH_3_C_6_H_4_	na	na	>200	nd
15	H	2-Naphthyl	na	na	>200	nd
16	C_6_H_5_	4-CH_3_C_6_H_4_	72.72 (58.20–90.87)	29.54 (27.68–31.52)	163.0 (143.2–185.7)	5.51
17	C_6_H_5_	4-OCH_3_C_6_H_4_	68.93 (62.24–76.33)	57.0 (52.01–62.46)	183.3 (48.09–698.7)	3.21
18	C_6_H_5_	4-OHC_6_H_4_	58.33 (50.84–66.91)	49.35 (44.66–54.53)	175.3 (81.14–378.9)	3.55
19	C_6_H_5_	4-NO_2_C_6_H_4_	9.06 (7.76–10.59)	na	69.51 (53.99–89.50)	nd
20	C_6_H_5_	3-CH_3_C_6_H_4_	26.43 (23.78–29.38)	14.70 (12.48–17.31)	137.6 (118.3–160.0)	9.36
21	C_6_H_5_	-C_6_H_5_	27.42 (24.53–30.65)	16.60 (14.28–19.29)	136.1 (115.4–160.4)	8.20
22	C_6_H_5_	2-CH_3_C_6_H_4_	30.94 (28.98–33.03)	25.43 (19.78–32.69)	45.11 (32.08–63.43)	1.77
23	C_6_H_5_	2-Naphthyl	27.52 (24.19–31.30)	46.89 (42.79–51.38)	152.7 (139.7–167.0)	3.25
24	C_6_H_5_	Propyl	na	na	>200	nd
25	4-NO_2_C_6_H_4_	4-CH_3_C_6_H_4_	107.8 (95.70–121.50)	na	42.65 (34.23–53.14)	nd
26	4-NO_2_C_6_H_4_	4-ClC_6_H_4_	49.17 (36.25–66.69)	na	33.89 (28.20–40.73)	nd
27	4-NO_2_C_6_H_4_	4-FC_6_H_4_	51.76 (27.65–96.90)	na	42.11 (24.33–72.90)	nd
28	4-NO_2_C_6_H_4_	-C_6_H_5_	40.08 (14.50–110.80)	na	94.18 (79.85–111.10)	nd
29	4-NO_2_C_6_H_4_	4-CH_3_SC_6_H_4_	20.35 (7.99–51.80)	39.15 (28.05–54.65)	75.32 (52.08–108.90)	1.92
30	4-NO_2_C_6_H_4_	4-NO_2_C_6_H_4_	120.5 (31.83–455.90)	45.80 (40.77–51.45)	81.56 (34.82–191.10)	1.78
31	4-NO_2_C_6_H_4_	4-OHC_6_H_4_	na	na	134.8 (71.86–253.00)	nd
32	4-NO_2_C_6_H_4_	4-OCH_3_C_6_H_4_	8.09 (3.55–18.46)	39.11 (35.41–43.19)	74.97 (45.40–123.80)	1.91
33	4-NO_2_C_6_H_4_	3-CH_3_C_6_H_4_	55.94 (26.68–117.30)	32.41 (30.62–34.31)	47.93 (42.61–53.91)	1.47
34	4-NO_2_C_6_H_4_	2-CH_3_C_6_H_4_	82.85 (45.93–149.50)	na	96.04 (88.29–104.50)	nd
35	4-NO_2_C_6_H_4_	Propyl	na	na	75.10 (54.27–103.90)	nd
36	4-NO_2_C_6_H_4_	2-Naphthyl	na	na	58.25 (34.63–98.00)	nd
miltefosine	-	-	16.85	17.80	122	6.85

na: not active; nd: not determined; IC_50_: 50% inhibitory concentration; CC_50_: 50% cytotoxic concentration; 95% C.I.: 95% confidence interval; S.I.: selectivity index (CC_50_ mammalian cells/IC_50_ amastigotes).

### Mammalian Cytotoxicity

The synthesized compounds were incubated with mammalian cells (NCTC clone 929) for 48 h to evaluate the *in vitro* cytotoxicity and the viability was detected by the colorimetric assay with MTT. Twenty-nine compounds showed 50% cytotoxic concentration (CC_50_) values in the range between 33.89 to 183.3 µM; compounds **9**, **10**, **11**, **13**, **14**, **15** and **24** showed lack of toxicity, and among these compounds, **10** and **11** showed activity against intracellular amastigotes. Miltefosine was used as standard drug and resulted in CC_50_ value of 122 µM.

### Analysis of Quantitative Structure-Activity Relationships (QSAR)

In order to gain information on relationships between the chemical structure and bioactivity, a QSAR analysis was undertaken with the activity data of twenty-seven compounds against *L.* (*L.*) *infantum* promastigotes. This set of activity data had to be chosen since the number of available data points for anti-amastigote activity and the variance within this set of data were limited.

QSAR aims at a mathematical description of the contributions of structural features and chemical properties, expressed in numerical form as descriptor variables, to bioactivity. Classically, linear regression methods are used to investigate the compounds for correlations between the descriptor and the biological activity data. Here, a set of 308 molecular descriptors were analyzed using a genetic algorithm as a means of variable selection for multiple linear regression (MLR) models, followed by analysis of the resulting QSAR equations of the best MLR models using partial least squares regression (PLS).

The best performance in PLS regression among all tested models was found with a QSAR equation consisting of four 3D descriptors. This model was characterized by an R^2^ (coefficient of determination for calibration vs experimental data) of 0.78 and a Q^2^ value (coefficient of determination for cross validation predictions vs. experimental data) of 0.68.

*QSAR equation (1):*

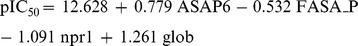
(R^2^ = 0.780, RMSE = 0.176, Q^2^ = 0.676, RMSE (cross validation) = 0.217; data were standardized to unit variance, i.e. each value was divided by the standard deviation of the descriptor column)

The descriptors encode the following properties: ASAP6 represents the accessible molecular surface area covered by atoms in a partial charge (*qi*) interval from +0.25 to +0.3 e [16] while FASA_P is the fraction of accessible molecular surface area covered by atoms with polar properties (i.e. |*qi*|≥0.2). ASAP6 within this series of compounds is mainly influenced by the partial charge and accessibility of the OH proton at the naphthoquinone core. In some compounds its calculated partial charge is below 0.25 e and apparently there is a correlation between activity and the degree of positive charge (or electron density) and accessibility of this acidic proton. Thus, it may be stated that the overall polarity of the molecules must not be too high for improving the antileishmanial activity (negative regression coefficient of FASA_P) while the OH proton in the quinone system has some influence, probably due to its H-bonding propensity (positive coefficient of ASAP6). Descriptors npr1 and glob are descriptors related to molecular mass distribution and shape. Npr1 is the normalized principal moment of inertia ratio pmi1/pmi3 where pmi1 and pmi3 are the first and third diagonal elements of the diagonalized moment of inertia tensor. The negative coefficient in the QSAR equation shows that a high ratio between these two elements would be detrimental to activity, i.e. that a more even mass distribution within the molecule has an enhancing effect on activity. Along the same lines, glob represents the molecular globularity, which is 1 in case of a perfect sphere and 0 in case of a purely 1 or 2-dimensional object. The positive regression coefficient in this case indicates that a more spherical shape appears to enhance activity in this series.

**Figure 2:**
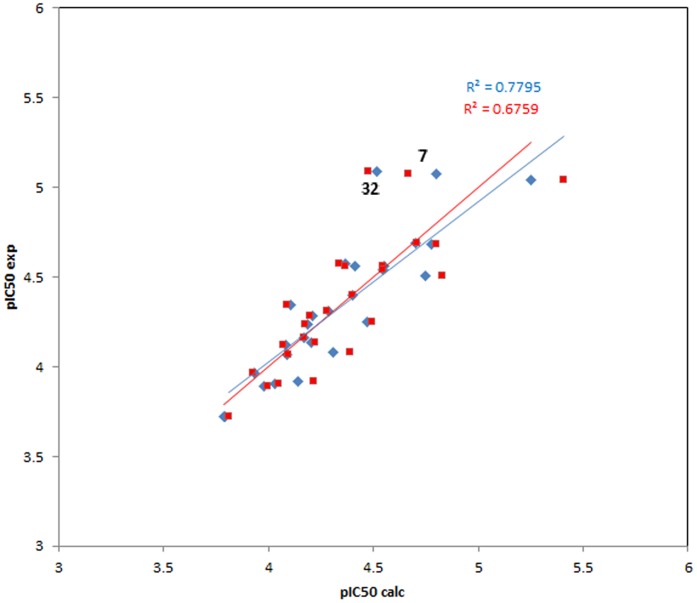
Plot of experimental pIC_50_ values vs. values calculated by QSAR equation (1). Blue rhombi: calibration data, red squares: leave one out cross validation predictions. Highlighted by ellipse: Data points for compounds **7** and **32** which are not well explained by the equation.

A plot of experimental data vs. calibration and cross validation data is shown in Figure 2. It becomes obvious in this plot that two compounds in the highest activity range, **32** and **7**, are not well represented by the model, i.e. their activity values calculated by the QSAR equation are much lower than the experimental ones. In fact, compound **32** causes the most dramatic effect on the overall correlation, since eliminating it increased the R^2^ and Q^2^ values to 0.85 and 0.75, respectively (omission of both, **32** and **7** yielded 0.85 and 0.75). Based on these data, it can safely be assumed that the QSAR model presented here explains the variance of the biological data for most of the compounds very well and thus captures the major structural influences on the bioactivity under study.

## Discussion

Naphthoquinones have been considered promising scaffolds against protozoan parasites. Atovaquone, a naphtoquinone, has been used as a fixed-dose combination with proguanil for treating children and adults with uncomplicated malaria or as chemoprophylaxis for preventing malaria in travellers [17]. Other naphtoquinones as buparvaquone (2-((4-tert-Butylcyclohexyl)methyl)-3-hydroxy-1,4-naphthoquinone), a veterinary drug against the protozoan parasite *Theileria* spp., has been found to be a promising lead against *Leishmania* spp, with IC_50_ values in the range of 0.005 and 0.12 µM against *L.* (*L.*) *donovani* [18]–[19]. The synthesis of 2-hydroxy-3-phenylsulfanylmethyl-[1,4]-naphthoquinones and their activity against the protozoan parasite *Plasmodium falciparum* was recently reported by Sharma and co-workers [9] and directed us to evaluate their potential *in vitro* activity against the etiologic agent of visceral leishmaniasis in Brazil and the Mediterranean region, the *L.* (*L.*) *infantum*.

In this study, 2-hydroxy-3-phenylsulfanylmethyl-[1,4]-naphthoquinones were synthesized taking into consideration that the naphthoquinone core remained the same in all the assayed compounds. Their IC_50_ values were evaluated against promastigotes and intracellular amastigotes of *L.* (*L.*) *infantum*. Among the thirty-six tested compounds, twenty-seven showed activity against the extracellular form of *Leishmania*, the promastigotes, but only compounds **7**, **10**, **19**, **29** and **32** showed similar effectiveness to the standard drug miltefosine. Considering the intracellular amastigotes as the clinically relevant forms, fourteen compounds showed activity; **10**, **11**, **16**, **20** and **21** were the most selective compounds, with selectivity index above 5. Taking into account the mammalian cytotoxicity, compound **11** was the most promising candidate; it was at least 2-fold more selective than miltefosine, showing a selectivity index higher than 15. Despite the clinical use of miltefosine, it has a considerable *in vitro* cytotoxicity to mammalian cells; in our assays, it showed a selectivity index of 6.

Corroborating the study of Sharma and co-workers [9], our most promising compounds also demonstrated the two phenyl groups at R_1_ and R_2_, but in contrast to *Plasmodium*, the antileishmanial activity was not enhanced when the *p*-nitro group was used as a substituent at R1. Furthermore, the presence of the two phenyl groups at R1 and R_2_ was mandatory to the anti-amastigote effect in our 2-hydroxy-3-phenylsulfanylmethyl-[1,4]-naphthoquinones, since the increased hydrophobicity possibly enhanced the penetration into macrophages or affected specific enzymes of the intracellular stage of the parasite. Although nitro compounds have shown promising antileishmanial activity [20] with IC_50_ values below 100 nM against intracellular amastigotes, their elevated toxicity has also been described in literature [21]. Our data corroborates previous reports, demonstrating a higher mammalian toxicity when the nitro group was introduced to our 2-hydroxy-3-phenylsulfanylmethyl-[1,4]-naphthoquinones.

Among the most active compounds containing the phenyl groups at R_1_ and R_2_, it is noteworthy that fluorine as a substituent at the phenyl ring at R_2_ (compound 11), decisively improved the selectivity (S.I.>15) towards intracellular amastigotes, also reducing the mammalian toxicity to undetectable levels at the highest tested concentration. In contrast, the substitution of fluorine by a hydroxyl group in the same position at R_2_ reduced at least 4-fold the selectivity of compound **18**. Despite the 3-fold increasing of the IC_50_ value, the substitution of fluorine (compound **11**) by chlorine (compound **10**) was also an important contribution, rendering a promising anti-amastigote activity with low mammalian toxicity. Both chlorine and fluorine possess some extreme properties, in particular, high electronegativity and oxidation potential, which may have contributed to the selectivity towards the intracellular amastigotes without affecting the host cell. Additionally, fluorine substitution can alter the chemical properties, disposition, and biological activity of drugs. Many fluorinated compounds are currently widely used in the treatment of diseases, including antimalarials, antibacterials, antifungals, antidepressants, antiinflammatory agents, antipsychotics, antivirals, steroids, and anesthetics [22]. Considering that fluorine is the most electronegative element in the periodic table, the replacement of a hydrogen atom for fluorine, can alter the pKa, the dipole moments, and even the chemical reactivity and stability of neighboring functional groups [23]. Sharma and co-workers [9] demonstrated the *in vitro* antimalarial activity against *P. falciparum* of a series of 2-hydroxy-3-phenylsulfanylmethyl-[1,4]-naphthoquinones, but conversely, the most active compounds (IC_50_<10 µM) had a *p*-nitro phenyl group as a substituent at R_1_.

Based on the substitution of methyl groups at different positions at R_2_, it was possible to note a considerable alteration in the biological effect. Changing the *p*-methyl group (compound **16**) to a *m*-methyl group (compound **20**) improved about 2-fold its anti-amastigote activity and its selectivity. On the other hand, the substitution of the *m*-methyl group (compound **20**) by the *o*-methyl group (compound **22**) increased about 3-fold the mammalian toxicity, while preserving the anti-amastigote activity at a similar level. Another important contribution could be observed by comparing compounds **16** and **17**; it is notable that the substitution of a methyl group at R_2_ (**16**) improved by 2-fold the anti-amastigote activity when compared to compound **17**, which displayed the methoxyl group at R_2_. Finally, the presence of the sulphur group at R_2_ (compound **12**) negatively contributed to the anti-amastigote effect when compared to compounds **16** and **17**.

Our synthesized series revealed moderate mammalian toxicity, as reflected by selectivity indexes ranging from 1.47 to >15. The cytotoxicity of naphthoquinones has been attributed to the induction of intrinsic apoptotic pathway in a manner associated with a significant reactive oxygen species (ROS) increase in the EL-4 mouse T lymphoma cells [24]. The capacity to produce free oxygen radicals is dramatically influenced by the nature and position of substituents and contributes to both therapeutic and toxic actions of these substances. The antitrypanosomal activity of other β-lapachol-derivatives has been attributed to the production of ROS and electrophilic metabolites, which bind to and inactivate *Trypanosoma cruzi* macromolecules [25]. Ribeiro and co-workers [26] also reported that pterocarpanquinone promotes apoptosis in *L.* (*L.*) *amazonensis* promastigotes through the formation of ROS, which cause oxidative stress, mitochondrial membrane depolarization and DNA fragmentation. It was found that diospyrin, a bisnaphthoquinonoid compound [1], and its derivative could induce apoptosis-like death in *L.* (*L.*) *donovani* promastigotes through depolarization of mitochondrial membrane potential [27]. Studies on the mechanism of antileishmanial activity showed diospyrin to be a specific inhibitor of type I DNA topoisomerase, an imperative therapeutic target for rational design of antiprotozoal drugs [28]. The antileishmanial activity of naphthoquinones against extracellular and intracellular *L.* (*L.*) *donovani* was also shown to be mediated by a nitric oxide-dependent mechanism [13].

Although the promastigote form is not the most relevant life stage with respect to the treatment of clinical infections, our QSAR analysis for the anti-promastigote activity reveals interesting general structural requirements for activity. The observation that descriptors of mass distribution and shape are of influence on the QSAR model indicates that the bioactivity under study depends on specific interactions with a particular biological target. Naphthoquinones may exert biological activity by mechanisms related to either oxidative modification or covalent interactions with target molecules. In the present case, due to the fact that the compounds do not have an unsubstituted carbon in the quinone ring, direct covalent reactions are unlikely. Quite interestingly, descriptors related to redox potential (i.e. HOMO and LUMO energies), which were also present in the variable set, did not yield significant contributions to the overall QSAR model. It may thus be assumed that the differences in activity observed between these molecules are not related to differences in the general mechanism of action but rather in subtle differences related to polarity, hydrogen-bonding and shape that influence non-covalent target binding.

It appears somewhat dissatisfactory that the two most active compounds in the series are not well described by the best QSAR equation we could find, which may indicate that these two compounds do not follow the common structure-activity relationship of the others. However, this may also mean that compounds **7** and **32** could act by a mechanism different from that of the other compounds of this series, which may be an interesting starting point for further studies. Compound **11**, the most selective compound against the intracellular amastigotes, on the other hand, is very well predicted in the QSAR model and thus seems to be mechanistically related (at least concerning the mechanism addressing the promastigotes) with the majority of the data set. However, amastigotes being the more relevant life stage for drug targeting, determination of IC_50_ values for a larger set of compounds would be useful in order to enable us to derive a QSAR model also for this life stage which would be desirable for further optimization of this promising class of antileishmanials.

Considering the *in vitro* antileshmanial potential of 2-hydroxy-3-phenylsulfanylmethyl-[1,4]-naphthoquinones and their structure-activity relationships, these compounds appear promising novel lead candidates that could be exploited in drug design studies for leishmaniasis.
